# Gender-specific sociodemographic and lifestyle factors associated with frailty status among Korean older adults

**DOI:** 10.1371/journal.pone.0348604

**Published:** 2026-05-08

**Authors:** Subeen Kim, Haerang Lee, Nayeon Park, Eunseo Choi, Minji Kang

**Affiliations:** 1 Department of Food and Nutrition, Duksung Women’s University, Seoul, Republic of Korea; 2 BNK Economic Research Institute, BNK Financial Group, Seoul, Republic of Korea; Yonsei University Medical Center: Yonsei University Health System, KOREA, REPUBLIC OF

## Abstract

This study aimed to identify gender-specific sociodemographic and lifestyle factors associated with frailty among Korean older adults. We conducted a cross-sectional analysis using data from the 2014–2019 Korean National Health and Nutrition Examination Survey, including 6,556 participants aged 65 years and older. Frailty was defined by the Fried phenotype. Multivariable logistic regression models were used to examine gender-specific associations between frailty and sociodemographic factors (age, education, marital status, household income, household type, and body mass index) as well as lifestyle factors (smoking, alcohol consumption, meals with family in the past year, energy intake, number of chronic diseases, and self-rated health status), with mutual adjustment for all listed covariates in a single fully adjusted model. The prevalence of frailty was 20.6% in women and 11.5% in men. Among women, current smoking [odds ratio (95% confidence interval), 2.22 (1.16–4.26)] and sharing meals with family in the morning [1.42 (1.05–1.93)] were associated with an increased risk of frailty. For men, high household income [0.51 (0.27–0.99)] and higher energy intake [>2,120 kcal; 0.49 (0.34–0.71)] were associated with a reduced risk of frailty. In both men and women, multiple chronic diseases and poor self-rated health were significantly associated with an increased risk of frailty. Frailty was associated with various sociodemographic and lifestyle characteristics in both men and women. The associations with several factors, such as smoking, meal patterns, and household income, differed by gender. This study highlights distinct gender-specific predictors of frailty among older Korean adults, emphasizing the need for tailored and integrated public health prevention strategies.

## Introduction

Korea is experiencing rapid population aging, with the proportion of individuals aged 65 and older reaching 19% as of 2024 [1]. This means that Korea is getting closer to entering a super-aged society according to the United Nations (UN) standard that classifies a society as an aging society if the elderly population ratio, which is the proportion of the population aged 65 or older, is 7% or more of the total population, an aged society if it is 14% or more, and a super-aged society if it is 20% or more [2]. Amid the rapid progression of population aging in Korea, this trend becomes even more pronounced when compared to other major developed countries. For example, as of 2024, Japan has the highest proportion of people aged 65 and older in the world at 29.3%, a figure projected to rise to 36.3% by 2045 [3]. In addition, Spain, which has a population size similar to that of Korea, is also expected to see its elderly population increase significantly from 17.4% in 2025 to 31.9% by 2049, indicating that population aging is intensifying not only in Korea but also in several other countries [1,4].

In light of these international trends, projections by Statistics Korea indicate that the proportion of the elderly population in Korea is expected to reach 29% by 2028 and 39.8% by 2049, surpassing the rate of aging observed in Japan. Furthermore, the aging process in Korea is progressing more rapidly than in Spain, despite their similar population sizes. These projections highlight that the proportion of individuals aged 65 and older in Korea will continue to increase sharply, underscoring the urgent need for heightened social awareness and policy measures concerning elderly health. In particular, health status is a major factor influencing the quality of life of the elderly, and therefore, preparations are needed to improve the health of the elderly in a super-aging society [5].

The increasing elderly population further emphasizes the need for research on frailty. Frailty is an age-related syndrome characterized by increased vulnerability to stressors and a decline in physiological reserves and physical function, which places individuals at higher risk for various diseases as well as a greater likelihood of functional dependence and hospitalization [6,7].

Traditionally, Research and policy discussions have often focused on the elderly population as a single group, paying relatively limited attention to heterogeneous characteristics and health needs that exist within this population. In recent years, a growing evidence has highlighted that frailty differs systematically between men and women [8,9]. Previous studies have consistently shown that the prevalence, trajectories, and predictors of frailty vary by sex, with women generally exhibiting a higher prevalence of frailty but lower mortality risk at a given level of frailty compared with men [10–12]. At the same time, even among men and women, there is considerable heterogeneity in frailty. Levels and patterns of frailty can differ according to socioeconomic position, living arrangements, nutritional status, and health behaviors, indication that sex alone is insufficient to capture the complexity of frailty in later life [8]. Sociological factors and lifestyle habits of older adults have been reported to significantly influence the development of frailty and are known to play an important role in preventing frailty and promoting healthy longevity [6]. A cross-sectional study indicated that older adults with frailty are approximately four times more likely to develop depression, while those with depression are four times more likely to become frail [13]. In addition, a study conducted in China recruited 12,227 participants aged 83.4 ± 11.0 years and investigated the effect of physical activity capacity on depression [14]. Participants with low physical activity capacity were 2.21 times more likely to suffer from depression than those with normal physical activity capacity [14]. These results suggest a close correlation between various risk factors and frailty, highlighting the growing emphasis on comprehensive approaches that integrate social contexts and individual lifestyle habits to support healthy aging through effective prevention and intervention strategies. Accordingly, frailty has increasingly been conceptualized not only as an individual clinical condition but also as an expression of accumulated social and structural disadvantage over the life course [15,16].

Building on previous studies, this study conducts sex-stratified analyses of frailty among older adults in Korea. Within each gender, the associations between frailty and a range of sociodemographic, nutritional, and health-behavioral factors—including educational attainment, household income, household type, body mass index, and alcohol consumption—are examined to more precisely characterize heterogeneity within the older population. Furthermore, rather than treating frailty as a homogeneous condition, this study simultaneously examines individual frailty components to identify subgroups that exhibit vulnerability in specific dimensions of frailty. Through this approach, the study aims to provide evidence to support the development of more targeted and effective strategies for frailty prevention and management among older adults.

## Materials and methods

### Study design and participants

This study used data from the Korean National Health and Nutrition Examination Survey (KNHANES) collected between 2014 and 2019. The KNHANES is an annual, nationwide cross-sectional survey conducted by the Korea Disease Control and Prevention Agency (KDCA), which uses a multi-stage stratified probability sampling method to achieve demographic representativeness. The study protocol was approved by the Institutional Review Board (IRB) of KDCA (approval numbers: 2009-01CON-03-2C, 2010-02CON-21-C, 2011-02CON-06-C, 2012-01EXP-01-2C, 2013-07CON-03-4C, 2013-12EXP-03-5C, 2018-01-03-P-A, 2018-01-03-C-A). In accordance with the bioethics and safety act, the study was exempted from IRB review in the years 2015, 2016, and 2017 [17].

**Fig 1:**
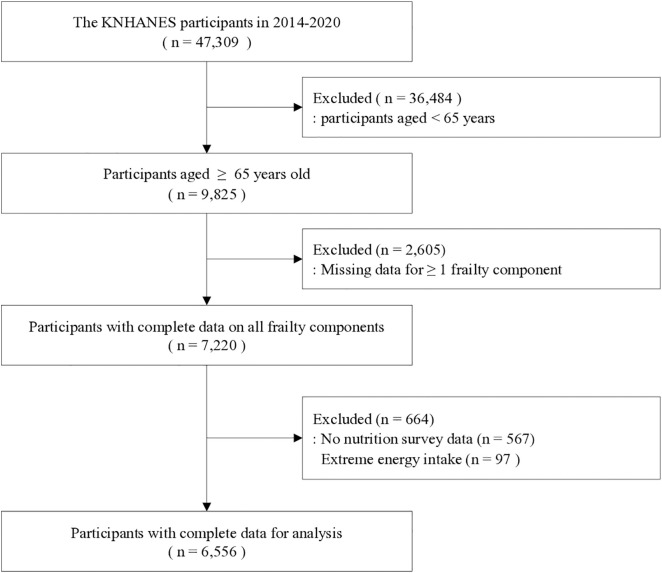
Flowchart of the study participants. KNHANES, Korea National Health and Nutrition Examination Survey.‌‌.

From 2014 to 2020, a total of 47,309 participants were included in the KNHANES. Among them, 9,825 individuals aged 65 years or older were identified. Participants with missing data for one or more frailty components were excluded (n = 2,605), leaving 7,220 participants with complete data on all frailty components. We further excluded those without nutrition survey data (n = 567) and those with extreme daily energy intake (< 500 kcal or > 5,000 kcal; n = 97). Consequently, the final analytic sample comprised 6,556 participants (men: 2,913, women: 3,643) (Fig 1).

### Sociodemographic characteristics

Sociodemographic variables included age, educational level, marital status, household income, and household type. Educational level was categorized as middle school or below, high school, or college or above. Marital status was classified as married/living with partner, separated/divorced/never married, or widowed. Separated, divorced, and never married participants were grouped together due to the small number of never married participants (n = 45). Household income was divided into four groups based on equivalized household income: low (lowest 25%), mid-low (26%–50%), mid-high (51%–75%), and high (highest 25%). Household type was classified as single-person or multi-person households.

### Lifestyle characteristics

Lifestyle factors included body mass index (BMI), smoking status, alcohol consumption, meals with family, number of chronic diseases, health status, and energy intake (kcal). BMI (kg/m²) was classified as underweight (BMI < 18.5), normal weight (18.5 ≤ BMI < 23), overweight (23 ≤ BMI < 25), or obese (BMI ≥ 25) [18]. Smoking status was categorized as never smoked, former smoker, or current smoker. Alcohol consumption was based on the frequency of alcohol consumption in the past year, categorized as “non-drinker” for those who did not drink at all, “low” for those who drank less than once a month or about once a month, “moderate” for those who drank 2–4 times a month or 2–3 times a week, and “frequent” for those who drank more than 4 times a week. Meals with family were determined based on participation in breakfast, lunch, or dinner with family during the previous year. The number of chronic diseases was categorized as 0, 1–2, or 3 or more, based on the presence of hypertension, stroke, myocardial infarction (or angina), osteoarthritis, rheumatoid arthritis, asthma, diabetes mellitus, cancer (stomach, liver, colon, breast, cervical, and lung), and liver disease (hepatitis B, hepatitis C, and cirrhosis). Health status was assessed by self-rated health and classified as good or poor. Average energy intake was assessed using a 24-hour dietary recall method, in which participants reported all foods and beverages consumed during the previous day.

### Assessment of frailty status

Assessment of frailty status was based on the modified Fried phenotype, utilizing five components adapted for the specific variables available in the KNHANES: unintended weight loss, exhaustion, weakness, slowness, and low physical activity, derived from items available in the KNHANES, as referenced in previous studies [7,19–22]. Due to the inherent design of the KNHANES dataset, which does not include the specific fatigue items from the Center for Epidemiological Studies Depression (CES-D) scale, exhaustion was evaluated using self-perceived stress as a proxy measure [20–23]. This substitution has been previously validated in Korean epidemiological studies, demonstrating robust construct validity through its significant associations with nutritional status and other health indicators [22,23]

The criteria for each component were as follows: (1) unintended weight loss, defined as a self-reported unintentional loss of more than 3 kg in the past year; (2) exhaustion, defined as a self-perceived high level of stress; (3) weakness, assessed as grip strength < 28 kg for men and < 18 kg for women according to the Asian Working Group for Sarcopenia; (4) slowness, defined as having a problem with walking or mobility according to the mobility domain of the Euro Quality of Life-5 Dimension questionnaire; and (5) low physical activity, defined as being in the lowest 20% of metabolic equivalent task (MET) scores by gender, calculated using the Global Physical Activity Questionnaire [21,23,24]. This 20% threshold was adopted to maintain consistency with Fried’s original methodology, which identified the most inactive quintile of the population, and has been extensively validated in Korean frailty research using KNHANES data [7,22,23]

Participants were classified as “frail” if they met three or more criteria, “pre-frail” if they met one or two criteria, and “robust” if they met none of the criteria. For analysis, participants were grouped into non-frail (pre-frail and robust) and frail groups.

### Statistical analyses

The KNHANES employs a complex sample design to ensure national representativeness. In this study, all analyses incorporated survey weights, stratification variables, and clustering to produce nationally representative estimates, with a gender-stratified approach applied throughout. Categorical variables were expressed as frequencies and percentages and analyzed using the Rao-Scott χ² test, while continuous variables were reported as mean ± standard error (SE) and evaluated by linear regression models. Multivariable logistic regression analysis was performed to examine associations between frailty status (non-frail vs frail) and its components with sociodemographic and lifestyle factors. For each predictor, a separate fully adjusted model was fitted with simultaneous adjustment for all listed covariates. Potential multicollinearity among predictors was assessed using variance inflation factors. All VIF values ranged from 1.06 to 2.04, indicating no evidence of problematic multicollinearity. Results are presented as odds ratios (ORs) with 95% confidence intervals (CIs).

Adjusted covariates included: age group (65–69, 70–74, 75–79, ≥80 years), education level (middle school or below, high school, college or above), marital status (married/living with partner, separated/divorced/never married, widowed), equivalized household income (low, mid-low, mid-high, high), household type (single-person, multi-person), BMI (underweight, normal, overweight, obese), smoking status (never, former, current), alcohol consumption in the past year (non-drinker (never/no), low (<1/month or 1/month), moderate (2–4/month or 2–3/week), frequent (≥4/week)), meals with family (breakfast, lunch, dinner), number of chronic diseases (0, 1–2, ≥3), self-rated health status (good, poor), and daily energy intake categorized into gender- specific tertiles. Gender-specific cut-off values were used to define tertiles: for men, <1573, 1573–2120, and >2120 kcal per day; for women, <1182, 1182–1615, and >1615 kcal per day.

Missing values in sociodemographic and lifestyle variables were imputed using the mode of each variable. The number of missing cases per variable were as follows: education level (n = 43), household income (n = 37), marital status (n = 3), BMI (n = 37), alcohol consumption (n = 2), meals with family (breakfast: n = 259, lunch: n = 275, dinner: n = 78), and self-rated health status (n = 1). Because the proportion of missing data was low for all covariates (<5% for each variable; breakfast 3.95%, lunch 4.19%, dinner 1.19%, and <1% for the remaining covariates), mode imputation was used to preserve sample size [25]. All statistical analyses were performed using SAS software (ver. 9.4, SAS Institute Inc., Cary, NC, USA) and the level of significance was set at P < 0.05.

## Results

The comparison of characteristics between genders is presented in Table 1. Of the total of 6,556 participants, 2,913 were men and 3,643 were women. Among men, 2,563 (88.5%) were classified as non-frail and 350 (11.5%) as frail, whereas among women, 2,867 (79.4%) were non-frail and 776 (20.6%) were frail. Compared to men, women were more likely to have received no formal education (17.6% vs. 5.6%), to be widowed (43.9% vs. 7.2%), and to live alone (24.8% vs. 10.0%). In addition, women had lower household income and a higher prevalence of obesity (P < .0001). Men were more likely to have ever smoked and to report moderate alcohol consumption in the past year, but were less likely to have chronic diseases (all P < .0001). Women were more likely to report poor self-rated health, not share meals with family members, and have a lower average energy intake compared to men (all P < .0001).

**Table 1:** Sociodemographic and lifestyle characteristics of study participants according to gender.

Characteristics	All		Men		Women	
(n = 6,556)		(n = 2,913)		(n = 3,643)	
**Demographic**						
Age group (years)						
65-69	2,174	(33.7)	986	(33.7)	1,188	(33.7)
70-74	1,860	(29.4)	844	(29.7)	1,016	(29.2)
75-79	1,500	(21.8)	657	(21.7)	843	(21.9)
≥80	1,022	(15.1)	426	(14.9)	596	(15.3)
**Socioeconomic**						
Education level						
Middle school or below	4,783	(70.5)	1,698	(57.0)	3,085	(82.2)
High school	1,116	(18.3)	730	(25.3)	386	(12.2)
College or above	657	(11.2)	485	(17.6)	172	(5.6)
Marital status						
Married/ living with partner	4,418	(67.9)	2,558	(88.2)	1,860	(50.3)
Separated/ divorced/ never married	340	(5.2)	146	(4.6)	194	(5.8)
Widowed	1,798	(26.9)	209	(7.2)	1,589	(43.9)
Household income^a^						
Low	2,986	(43.5)	1,131	(37.7)	1,855	(48.5)
Middle-low	1,865	(28.0)	930	(31.3)	935	(25.2)
Middle-high	1,024	(16.8)	508	(18.0)	516	(15.8)
High	681	(11.6)	344	(13.0)	337	(10.5)
Household type						
Single-person	1,452	(18.0)	345	(10.0)	1,107	(24.8)
Multi-person	5,104	(82.0)	2,568	(90.0)	2,536	(75.2)
**Anthropometric**						
Body mass index						
Underweight	160	(2.4)	89	(3.0)	71	(1.8)
Normal	2,185	(33.4)	1,032	(34.9)	1,153	(32.2)
Overweight	1,789	(27.8)	840	(29.6)	949	(26.2)
Obese	2,422	(36.4)	952	(32.5)	1,470	(39.8)
**Health behaviors**						
Smoking status						
Never smokers	4,034	(60.1)	591	(20.5)	3,443	(94.2)
Former-smokers	1,936	(30.6)	1,809	(62.1)	127	(3.6)
Current smokers	586	(9.3)	513	(17.5)	73	(2.3)
Alcohol consumption (past year)						
Non-drinker (never/no)	3,168	(47.0)	932	(31.3)	2,236	(60.5)
Low (<1/month or 1/month)	1,480	(22.8)	505	(17.2)	975	(27.6)
Moderate (2–4/month or 2–3/week)	1,380	(21.9)	1,012	(35.4)	368	(10.3)
Frequent (≥4/week)	528	(8.3)	464	(16.1)	64	(1.6)
Meals with family in the past year (yes)						
Breakfast	4,216	(63.9)	2,146	(71.7)	2,070	(57.1)
Lunch	3,975	(59.5)	2,000	(66.9)	1,975	(53.1)
Dinner	4,465	(68.4)	2,294	(77.9)	2,171	(60.1)
Energy intake (kcal)	1684.6 ± 11.6		1924.9 ± 16.5		1477.7 ± 12.2	
**Health status**						
Number of chronic disease(s)^b^						
0	1,497	(23.6)	813	(28.1)	684	(19.8)
1-2	4,182	(63.4)	1,835	(63.1)	2,347	(63.6)
≥3	877	(13.0)	265	(8.8)	612	(16.6)
Self-rated health status						
Good	1,495	(23.1)	835	(28.4)	660	(18.6)
Poor	5,061	(76.9)	2,078	(71.6)	2,983	(81.4)
**Frailty**						
Frailty status						
Non-frail	5,430	(83.6)	2,563	(88.5)	2,867	(79.4)
Frail	1,126	(16.4)	350	(11.5)	776	(20.6)
Components of frailty (yes)						
Unintended weight loss	500	(7.5)	201	(6.6)	299	(8.3)
Weakness	2,572	(38.5)	929	(31.6)	1,643	(55.5)
Slowness	2,342	(34.3)	759	(25.3)	1,583	(42.0)
Exhaustion	223	(3.2)	63	(2.0)	160	(4.2)
Low physical activity	3,721	(55.4)	1,607	(53.4)	2,114	(57.1)

Values are n (%) or mean ± standard error. Significant differences in all distributions and mean values were found between men and women (P < 0.05), except for age group (P = 0.9681).

^a^Household income was categorized into quartiles based on equalized household income: low (lowest 25%), middle-low (26%−50%), middle-high (51%−75%), and high (highest 25%).

^b^Chronic diseases include hypertension, stroke, myocardial infarction, angina, osteoarthritis, rheumatoid arthritis, asthma, diabetes mellitus, cancer, and liver disease.

The gender-specific characteristics according to frailty status are presented in Table 2. Higher age showed a positive association with frailty prevalence in both genders, lower educational attainment and household income demonstrated significant inverse associations with frailty rates (all P < .0001). Similarly, average daily energy intake decreased as frailty status worsened. However, in terms of BMI, frail men were predominantly of normal weight (36.1%) or overweight (28.1%), whereas a higher proportion of frail women were classified as obese (42.3%). Most women were never smokers (711, 90.7%), and this association was statistically significant (P = 0.0007). In contrast, the majority of men had smoked at least once, but this was not statistically significant (P = 0.125). For alcohol consumption, the proportion of non-drinkers was higher in the frail group for both men and women. Among frail participants, the proportion of frequent drinkers was higher in men than in women (20.3% vs. 1.2%). Furthermore, the prevalence of frailty was higher among participants with three or more chronic diseases or those who perceived their health status as poor.

**Table 2:** Sociodemographic and lifestyle factors of study participants according to frailty status by gender.

Characteristics	Men (n = 2,913)					Women (n = 3,643)				
Non-frail (n = 2,563)		Frail (n = 350)		P-value	Non-frail (n = 2,867)		Frail (n = 776)		P-value
**Demographic**										
Age group (years)					<.0001					<.0001
65-69	942	(36.6)	44	(12.2)		1,068	(38.1)	120	(16.7)	
70-74	763	(30.7)	81	(21.8)		853	(30.9)	163	(22.8)	
75-79	550	(20.6)	107	(29.9)		614	(20.3)	229	(27.8)	
≥80	308	(12.1)	118	(36.1)		332	(10.7)	264	(32.7)	
**Socioeconomic**										
Education level					<.0001					<.0001
Middle school or below	1,431	(54.7)	267	(74.9)		2,345	(79.1)	740	(94.1)	
High school	676	(26.7)	54	(15.1)		364	(14.5)	22	(3.4)	
College or above	456	(18.6)	29	(10.0)		158	(6.5)	14	(2.5)	
Marital status					<.0001					<.0001
Married/ living with partner	2,267	(89.2)	291	(80.7)		1,541	(52.7)	319	(41.1)	
Separated/ divorced/ never married	126	(4.5)	20	(5.0)		145	(5.4)	49	(7.0)	
Widowed	170	(6.3)	39	(14.3)		1,181	(41.8)	408	(51.9)	
Household income^a^					<.0001					<.0001
Low	919	(35.1)	212	(57.5)		1,344	(44.8)	511	(63.0)	
Middle-low	841	(31.9)	89	(26.7)		787	(26.6)	148	(19.9)	
Middle-high	474	(19.0)	34	(10.8)		449	(17.3)	67	(9.9)	
High	329	(14.0)	15	(5.0)		287	(11.3)	50	(7.2)	
Household type					0.4605					0.0001
Single-person	302	(9.9)	43	(11.2)		821	(23.3)	286	(30.5)	
Multi-person	2,261	(90.1)	307	(88.8)		2,046	(76.7)	490	(69.5)	
**Anthropometric**										
Body mass index					<.0001					0.0012
Underweight	57	(2.3)	32	(7.9)		47	(1.4)	24	(3.6)	
Normal	899	(34.8)	133	(36.1)		919	(32.8)	234	(30.0)	
Overweight	749	(29.8)	91	(28.1)		766	(26.7)	183	(24.2)	
Obese	858	(33.1)	94	(28.0)		1,135	(39.1)	335	(42.3)	
**Health behaviors**										
Smoking status					0.125					0.0007
Never smokers	533	(20.9)	58	(17.4)		2,732	(95.1)	711	(90.7)	
Former-smokers	1,595	(62.2)	214	(60.9)		87	(3.0)	40	(5.6)	
Current smokers	435	(16.9)	78	(21.7)		48	(1.9)	25	(3.7)	
Alcohol consumption (past year)					<.0001					0.0006
Non-drinker (never/no)	771	(29.5)	161	(45.0)		1,688	(58.6)	548	(67.8)	
Low (<1/month or 1/month)	458	(17.9)	47	(11.9)		819	(28.9)	156	(22.8)	
Moderate (2–4/month or 2–3/week)	940	(37.1)	72	(22.8)		306	(10.8)	62	(8.2)	
Frequent (≥4/week)	394	(15.5)	70	(20.3)		54	(1.7)	10	(1.2)	
Meals with family in the past year (yes)										
Breakfast	1,885	(72.2)	261	(67.9)	0.1876	1,649	(57.8)	421	(54.4)	0.167
Lunch	1,749	(66.7)	251	(68.5)	0.57	1,565	(53.5)	410	(51.5)	0.4283
Dinner	2,024	(78.7)	270	(71.8)	0.0129	1,761	(62.1)	410	(52.6)	<.0001
Energy intake (kcal)	1962.7 ± 17.5		1635.4 ± 34.3		<.0001	1512.5 ± 13.7		1343.5 ± 22.8		<.0001
**Health status**										
No. of chronic disease(s)^b^					<.0001					<.0001
0	750	(29.2)	63	(19.2)		588	(21.7)	96	(12.4)	
1-2	1,614	(63.4)	221	(61.0)		1,846	(63.3)	501	(65.0)	
≥3	199	(7.4)	66	(19.7)		433	(15.0)	179	−22.6	
Self-rated health status					<.0001					<.0001
Good	799	(30.8)	36	(10.2)		595	(21.2)	65	(8.5)	
Poor	1,764	(69.2)	314	(89.8)		2,272	(78.8)	711	(91.5)	
Components of frailty (yes)										
Unintended weight loss	104	(3.9)	97	(27.7)	<.0001	112	(4.1)	187	(24.5)	<.0001
Weakness	607	(23.6)	322	(92.6)	<.0001	933	(32.2)	710	(91.5)	<.0001
Slowness	453	(17.1)	306	(88.3)	<.0001	871	(29.1)	712	(91.7)	<.0001
Exhaustion	32	(1.3)	31	(7.6)	<.0001	58	(1.9)	102	(12.8)	<.0001
Low physical activity	1,263	(47.5)	344	(98.4)	<.0001	1,367	(47.0)	747	(96.1)	<.0001

Values are n (%) or mean ± standard erros.

^a^Household income was categorized into quartiles based on equalized household income: low (lowest 25%), middle-low (26%−50%), middle-high (51%−75%), and high (highest 25%).

^b^Chronic diseases include hypertension, stroke, myocardial infarction, angina, osteoarthritis, rheumatoid arthritis, asthma, diabetes mellitus, cancer, and liver disease.

Sociodemographic and lifestyle factors associated with frailty status showed different patterns by gender (Table 3). The risk of frailty increased with age, with both men and women aged 80 years or older showing the highest risk (men: OR = 5.58, 95% CI: 3.37–9.22; women: OR = 5.41, 95% CI: 3.85–7.60). Attaining a high school level of education was associated with lower odds of frailty in both men (OR = 0.58, 95% CI: 0.40–0.84) and women (OR = 0.30, 95% CI: 0.17–0.51). In addition, higher household income tended to be associated with a reduced risk of frailty. Specifically, men in the highest income group (OR = 0.51, 95% CI: 0.27–0.99) and women in both the lower-middle (OR = 0.75, 95% CI: 0.57–0.97) and upper-middle income groups (OR = 0.61, 95% CI: 0.42–0.87) had lower odds of frailty compared with the lowest income group. Household type was associated with frailty only in men; compared with single-person households, multi-person households showed higher odds of frailty (OR = 2.12, 95% CI: 1.03–4.35).

**Table 3:** Sociodemographic and lifestyle factors associated with frailty by gender.

Variable	Frail	
**Men (n = 2,913)**	**Women (n = 3,643)**
**Demographic**		
Age group (years)		
65-69	1	1
70-74	1.63 (1.03 - 2.56)	1.39 (1.02 - 1.90)
75-79	2.87 (1.77 - 4.66)	2.39 (1.73 - 3.29)
≥80	5.58 (3.37 - 9.22)	5.41 (3.85 - 7.60)
**Socioeconomic**		
Education level		
Middle school or below	1	1
High school	0.58 (0.40 - 0.84)	0.30 (0.17 - 0.51)
College or above	0.67 (0.40 - 1.14)	0.59 (0.30 - 1.16)
Marital status		
Married/ living with partner	1	1
Separated/ divorced/ never married	1.70 (0.78 - 3.70)	1.53 (0.92 - 2.53)
Widowed	1.67 (0.89 - 3.14)	0.84 (0.62 - 1.14)
Household income^a^		
Low	1	1
Middle-low	0.85 (0.60 - 1.19)	0.75 (0.57 - 0.97)
Middle-high	0.70 (0.40 - 1.23)	0.61 (0.42 - 0.87)
High	0.51 (0.27 - 0.99)	0.76 (0.51 - 1.15)
Household type		
Single-person	1	1
Multi-person	2.12 (1.03 - 4.35)	1.17 (0.86 - 1.60)
**Anthropometric**		
Body mass index		
Underweight	1	1
Normal	0.38 (0.19 - 0.74)	0.48 (0.27 - 0.85)
Overweight	0.38 (0.19 - 0.77)	0.44 (0.25 - 0.77)
Obese	0.33 (0.16 - 0.67)	0.51 (0.29 - 0.90)
**Health behaviors**		
Smoking status		
Never smokers	1	1
Former-smokers	1.13 (0.76 - 1.69)	1.60 (0.98 - 2.61)
Current smokers	1.55 (0.95 - 2.51)	2.22 (1.16 - 4.26)
Alcohol consumption (past year)		
Non-drinker (never/no)	1	1
Low (<1/month or 1/month)	0.63 (0.40 - 0.99)	0.91 (0.72 - 1.15)
Moderate (2–4/month or 2–3/week)	0.65 (0.44 - 0.95)	0.82 (0.58 - 1.15)
Frequent (≥4/week)	1.27 (0.84 - 1.92)	0.58 (0.27 - 1.26)
Meals with family in the past year		
Breakfast		
No	1	1
Yes	0.85 (0.54 - 1.34)	1.42 (1.05 - 1.93)
Lunch		
No	1	1
Yes	1.29 (0.89 - 1.86)	1.01 (0.79 - 1.29)
Dinner		
No	1	1
Yes	0.65 (0.41 - 1.03)	0.71 (0.52 - 0.96)
Energy intake^b^		
Tertile 1	1	1
Tertile 2	0.71 (0.51 - 0.99)	0.85 (0.67 - 1.07)
Tertile 3	0.49 (0.34 - 0.71)	0.73 (0.56 - 0.94)
**Health status**		
No. of chronic disease(s)^c^		
0	1	1
1-2	1.32 (0.90 - 1.93)	1.33 (0.99 - 1.78)
≥3	3.60 (2.18 - 5.94)	1.67 (1.16 - 2.41)
Self-rated health status		
Good	1	1
Poor	2.61 (1.69 - 4.02)	2.36 (1.71 - 3.27)

^a^Household income was categorized into quartiles based on equalized household income: low (lowest 25%), middle-low (26%−50%), middle-high (51%−75%), and high (highest 25%).

^b^Energy intake tertiles were defined using gender-specific cut-off values: for men, Tertile 1 < 1,573 kcal/day, Tertile 2 1,573–2,120 kcal/day, and Tertile 3 > 2,120 kcal/day; for women, Tertile 1 < 1,182 kcal/day, Tertile 2 1,182–1,615 kcal/day, and Tertile 3 > 1,615 kcal/day.

^c^Chronic diseases include hypertension, stroke, myocardial infarction, angina, osteoarthritis, rheumatoid arthritis, asthma, diabetes mellitus, cancer, and liver disease.

Odds ratios and 95% confidence intervals were calculated by comparing the frail group to the non-frail group (reference).

Multivariable logistic regression was performed in participants with complete information on covariates (2,913 men and 3,643 women).

Estimates for each factor (e.g., age group, education level) were adjusted for all other factors in the table. Analyses were repeated separately for each factor.

BMI was associated with frailty; using underweight as the reference, both men and women in the normal weight, overweight, and obese categories showed lower odds of frailty (Table 3). Smoking status and alcohol consumption exhibited showed different patterns by gender. Among women, current smoking was associated with higher odds of frailty (OR = 2.22, 95% CI: 1.16–4.26). For alcohol consumption, compared with non-drinkers, men with low and moderate alcohol intake showed lower odds of frailty (low: OR = 0.63, 95% CI: 0.40–0.99; moderate: OR = 0.65, 95% CI: 0.44–0.95).

The frequency of family meals over the past year showed contrasting results according to gender and meal timing. Among women, eating breakfast with family was associated with a higher risk of frailty (OR = 1.42, 95% CI: 1.05–1.93), whereas eating dinner with family was associated with a lower risk of frailty (OR = 0.71, 95% CI: 0.53–0.96). Regarding daily energy intake categorized into gender-specific tertiles, older adults in the highest intake group had lower risk of frailty in both men (OR = 0.49, 95% CI: 0.34–0.71) and women (OR = 0.73, 95% CI: 0.56–0.94). Having three or more chronic diseases was associated with a greater increase in frailty risk in men (OR = 3.60, 95% CI: 2.18–5.94) than in women (OR = 1.67, 95% CI: 1.16–2.41). Poor self-rated health was associated with higher odds of frailty in both men (OR = 2.61, 95% CI: 1.69–4.02) and women (OR = 2.36, 95% CI: 1.71–3.27) (Table 3).

In addition to examining overall frailty, we conducted further analyses to assess whether, within each gender, individual frailty components were differentially associated with sociodemographic and lifestyle factors. This approach helps to identify which functional domains of frailty are particularly affected in specific subgroups and may inform the development of more tailored prevention and management strategies. Sociodemographic and lifestyle factors according to each frailty component by gender are presented in Table 4 and Table 5.

**Table 4:** Sociodemographic and lifestyle factors associated with components of frailty in men.

	Men (n = 2,913)				
Components of Frailty				
Variable	Weight loss	Weakness	Slowness	Exhaustion	Low PA
**Demographic**					
Age group (years)					
65-69	1	1	1	1	1
70-74	0.85 (0.54 - 1.33)	2.00 (1.50 - 2.66)	1.21 (0.91 - 1.62)	0.38 (0.17 - 0.85)	1.11 (0.89 - 1.37)
75-79	1.09 (0.66 - 1.80)	3.12 (2.34 - 4.17)	1.98 (1.44 - 2.70)	0.26 (0.11 - 0.62)	1.33 (1.03 - 1.71)
≥80	1.12 (0.63 - 1.98)	8.80 (6.32 - 12.24)	3.68 (2.62 - 5.18)	0.47 (0.20 - 1.12)	1.85 (1.38 - 2.49)
**Socioeconomic**					
Education level					
Middle school or below	1	1	1	1	1
High school	0.72 (0.45 - 1.16)	0.57 (0.44 - 0.74)	0.66 (0.51 - 0.85)	0.61 (0.28 - 1.36)	0.64 (0.52 - 0.79)
College or above	0.60 (0.33 - 1.10)	0.65 (0.47 - 0.90)	0.58 (0.42 - 0.82)	0.69 (0.27 - 1.72)	0.62 (0.47 - 0.82)
Marital status					
Married/ living with partner	1	1	1	1	1
Separated/ divorced/ never married	1.60 (0.63 - 4.11)	0.94 (0.55 - 1.63)	1.31 (0.75 - 2.29)	1.22 (0.42 - 3.54)	1.15 (0.69 - 1.93)
Widowed	0.89 (0.38 - 2.11)	1.22 (0.73 - 2.03)	1.44 (0.87 - 2.39)	2.16 (0.71 - 6.54)	1.32 (0.86 - 2.02)
Household income^a^					
Low	1	1	1	1	1
Middle-low	1.22 (0.79 - 1.88)	0.65 (0.51 - 0.83)	0.97 (0.76 - 1.25)	0.88 (0.43 - 1.77)	1.19 (0.95 - 1.50)
Middle-high	0.86 (0.46 - 1.60)	0.76 (0.54 - 1.07)	0.82 (0.60 - 1.13)	1.19 (0.45 - 3.15)	1.18 (0.90 - 1.55)
High	0.48 (0.18 - 1.26)	0.67 (0.46 - 0.99)	0.57 (0.38 - 0.86)	0.80 (0.30 - 2.12)	1.08 (0.77 - 1.52)
Household type					
Single-person	1	1	1	1	1
Multi-person	2.50 (1.02 - 6.13)	1.23 (0.74 - 2.07)	1.23 (0.78 - 1.97)	0.61 (0.23 - 1.62)	1.06 (0.70 - 1.59)
**Anthropometric**					
Body mass index					
Underweight	1	1	1	1	1
Normal	0.32 (0.18 - 0.58)	0.37 (0.21 - 0.65)	0.69 (0.38 - 1.25)	1.07 (0.18 - 6.47)	0.79 (0.45 - 1.40)
Overweight	0.20 (0.11 - 0.38)	0.27 (0.15 - 0.48)	1.02 (0.56 - 1.86)	0.60 (0.10 - 3.72)	0.69 (0.39 - 1.24)
Obese	0.15 (0.08 - 0.30)	0.22 (0.12 - 0.39)	1.17 (0.65 - 2.12)	1.14 (0.19 - 6.81)	0.74 (0.42 - 1.31)
**Health behaviors**					
Smoking status					
Never smokers	1	1	1	1	1
Former-smokers	0.99 (0.62 - 1.59)	0.92 (0.71 - 1.19)	1.12 (0.84 - 1.48)	1.19 (0.52 - 2.73)	1.17 (0.92 - 1.47)
Current smokers	1.56 (0.95 - 2.56)	1.02 (0.73 - 1.42)	1.33 (0.92 - 1.92)	1.59 (0.62 - 4.12)	1.50 (1.12 - 2.00)
Alcohol consumption (past year)					
Non-drinker (never/no)	1	1	1	1	1
Low (<1/month or 1/month)	0.91 (0.53 - 1.54)	0.84 (0.62 - 1.14)	0.70 (0.51 - 0.96)	1.19 (0.46 - 3.12)	0.91 (0.70 - 1.17)
Moderate (2–4/month or 2–3/week)	0.66 (0.42 - 1.03)	0.60 (0.46 - 0.77)	0.69 (0.54 - 0.89)	1.21 (0.57 - 2.59)	0.77 (0.62 - 0.97)
Frequent (≥4/week)	0.73 (0.43 - 1.26)	1.11 (0.83 - 1.49)	0.85 (0.63 - 1.15)	1.52 (0.73 - 3.13)	0.85 (0.65 - 1.11)
Meals with family in the past year					
Breakfast					
No	1	1	1	1	1
Yes	0.98 (0.62 - 1.54)	0.73 (0.54 - 0.98)	0.87 (0.63 - 1.20)	0.52 (0.26 - 1.05)	1.06 (0.83 - 1.36)
Lunch					
No	1	1	1	1	1
Yes	1.21 (0.76 - 1.92)	1.24 (0.94 - 1.62)	1.03 (0.80 - 1.33)	1.30 (0.65 - 2.62)	1.01 (0.82 - 1.25)
Dinner					
No	1	1	1	1	1
Yes	0.44 (0.28 - 0.71)	0.83 (0.59 - 1.17)	0.98 (0.69 - 1.40)	1.23 (0.52 - 2.94)	1.00 (0.74 - 1.34)
Energy intake^b^					
Tertile 1	1	1	1	1	1
Tertile 2	0.81 (0.54 - 1.22)	0.75 (0.59 - 0.96)	0.91 (0.71 - 1.17)	1.17 (0.58 - 2.36)	0.97 (0.79 - 1.19)
Tertile 3	0.75 (0.45 - 1.25)	0.63 (0.49 - 0.83)	0.89 (0.67 - 1.17)	0.72 (0.34 - 1.52)	0.73 (0.59 - 0.92)
**Health status**					
No. of chronic disease(s)^c^					
0	1	1	1	1	1
1-2	1.24 (0.84 - 1.84)	1.12 (0.88 - 1.42)	1.57 (1.18 - 2.08)	0.98 (0.52 - 1.86)	1.25 (1.01 - 1.53)
≥3	1.69 (0.87 - 3.27)	1.78 (1.21 - 2.62)	4.32 (2.86 - 6.55)	1.12 (0.35 - 3.58)	1.88 (1.30 - 2.70)
Self-rated health status					
Good	1	1	1	1	1
Poor	1.46 (0.88 - 2.42)	1.15 (0.89 - 1.47)	3.51 (2.59 - 4.75)	1.63 (0.75 - 3.53)	1.37 (1.13 - 1.67)

^a^Household income was categorized into quartiles based on equalized household income: low (lowest 25%), middle-low (26%−50%), middle-high (51%−75%), and high (highest 25%).

^b^Energy intake tertiles were defined using gender-specific cut-off values: for men, Tertile 1 < 1,573 kcal/day, Tertile 2 1,573–2,120 kcal/day, and Tertile 3 > 2,120 kcal/day; for women, Tertile 1 < 1,182 kcal/day, Tertile 2 1,182–1,615 kcal/day, and Tertile 3 > 1,615 kcal/day.

^c^Chronic diseases include hypertension, stroke, myocardial infarction, angina, osteoarthritis, rheumatoid arthritis, asthma, diabetes mellitus, cancer, and liver disease.

Odds ratios and 95% confidence intervals were calculated by comparing participants with each frailty component to those without the respective component (reference).

Multivariable logistic regression was performed in participants with complete information on covariates (2,913 men).

Estimates for each factor (e.g., age group, education level) were adjusted for all other factors in the table. Analyses were repeated separately for each factor.

**Table 5:** Sociodemographic and lifestyle factors associated with components of frailty in women.

	Women (n = 3,643)				
Components of Frailty				
Variable	Weight loss	Weakness	Slowness	Exhaustion	Low PA
**Demographic**					
Age group (years)					
65-69	1	1	1	1	1
70-74	1.09 (0.71 - 1.66)	1.61 (1.30 - 2.01)	1.42 (1.14 - 1.78)	0.89 (0.55 - 1.43)	1.23 (0.99 - 1.52)
75-79	1.57 (1.04 - 2.37)	3.14 (2.50 - 3.95)	2.15 (1.68 - 2.76)	0.92 (0.53 - 1.58)	1.76 (1.40 - 2.22)
≥80	2.22 (1.41 - 3.48)	7.87 (5.84 - 10.61)	2.82 (2.12 - 3.76)	0.70 (0.36 - 1.37)	2.90 (2.20 - 3.80)
**Socioeconomic**					
Education level					
Middle school or below	1	1	1	1	1
High school	0.47 (0.26 - 0.86)	0.61 (0.46 - 0.82)	0.44 (0.32 - 0.60)	0.27 (0.11 - 0.71)	0.81 (0.62 - 1.06)
College or above	0.48 (0.13 - 1.85)	0.95 (0.63 - 1.44)	0.61 (0.39 - 0.97)	0.43 (0.14 - 1.37)	0.72 (0.50 - 1.04)
Marital status					
Married/ living with partner	1	1	1	1	1
Separated/ divorced/ never married	0.75 (0.36 - 1.57)	1.31 (0.85 - 2.01)	1.52 (1.00 - 2.31)	0.51 (0.21 - 1.23)	0.97 (0.65 - 1.44)
Widowed	0.95 (0.61 - 1.49)	1.00 (0.78 - 1.28)	0.89 (0.69 - 1.15)	0.33 (0.20 - 0.56)	0.82 (0.64 - 1.04)
Household income^a^					
Low	1	1	1	1	1
Middle-low	0.82 (0.56 - 1.19)	0.82 (0.67 - 1.00)	0.84 (0.68 - 1.03)	0.91 (0.60 - 1.38)	0.78 (0.64 - 0.96)
Middle-high	1.02 (0.62 - 1.69)	0.84 (0.64 - 1.11)	0.74 (0.56 - 0.96)	0.48 (0.23 - 1.03)	0.65 (0.50 - 0.84)
High	1.01 (0.62 - 1.66)	0.91 (0.66 - 1.25)	0.76 (0.55 - 1.05)	0.71 (0.34 - 1.49)	0.77 (0.58 - 1.02)
Household type					
Single-person	1	1	1	1	1
Multi-person	0.91 (0.59 - 1.42)	1.12 (0.86 - 1.46)	1.15 (0.89 - 1.51)	1.57 (0.83 - 3.00)	1.00 (0.78 - 1.27)
**Anthropometric**					
Body mass index					
Underweight	1	1	1	1	1
Normal	1.07 (0.44 - 2.61)	0.33 (0.19 - 0.60)	0.68 (0.39 - 1.19)	0.45 (0.13 - 1.51)	0.77 (0.45 - 1.31)
Overweight	0.95 (0.39 - 2.29)	0.26 (0.14 - 0.48)	0.68 (0.39 - 1.20)	0.38 (0.12 - 1.25)	0.88 (0.51 - 1.51)
Obese	0.44 (0.18 - 1.06)	0.23 (0.13 - 0.42)	1.15 (0.66 - 1.99)	0.46 (0.13 - 1.57)	0.90 (0.53 - 1.53)
**Health behaviors**					
Smoking status					
Never smokers	1	1	1	1	1
Former-smokers	1.67 (0.84 - 3.32)	0.80 (0.50 - 1.28)	1.47 (0.95 - 2.29)	1.51 (0.64 - 3.57)	1.37 (0.91 - 2.08)
Current smokers	0.89 (0.38 - 2.09)	1.01 (0.57 - 1.78)	1.98 (1.08 - 3.62)	1.92 (0.70 - 5.24)	1.50 (0.80 - 2.82)
Alcohol consumption (past year)					
Non-drinker (never/no)	1	1	1	1	1
Low (<1/month or 1/month)	0.91 (0.64 - 1.29)	0.75 (0.61 - 0.92)	1.09 (0.90 - 1.31)	0.47 (0.29 - 0.78)	1.08 (0.89 - 1.30)
Moderate (2–4/month or 2–3/week)	1.08 (0.66 - 1.78)	0.92 (0.69 - 1.22)	1.03 (0.79 - 1.35)	0.80 (0.42 - 1.56)	0.89 (0.68 - 1.16)
Frequent (≥4/week)	0.59 (0.20 - 1.71)	0.68 (0.36 - 1.27)	0.68 (0.37 - 1.25)	2.16 (0.86 - 5.44)	1.12 (0.63 - 1.98)
Meals with family in the past year					
Breakfast					
No	1	1	1	1	1
Yes	1.15 (0.72 - 1.85)	0.96 (0.75 - 1.23)	1.02 (0.79 - 1.32)	1.22 (0.74 - 2.01)	1.29 (1.02 - 1.63)
Lunch					
No	1	1	1	1	1
Yes	1.12 (0.81 - 1.55)	0.89 (0.73 - 1.08)	1.04 (0.86 - 1.26)	0.83 (0.56 - 1.23)	0.90 (0.75 - 1.08)
Dinner					
No	1	1	1	1	1
Yes	1.05 (0.66 - 1.69)	1.16 (0.90 - 1.50)	0.81 (0.62 - 1.05)	0.41 (0.25 - 0.66)	0.84 (0.65 - 1.09)
Energy intake^b^					
Tertile 1	1	1	1	1	1
Tertile 2	1.11 (0.81 - 1.54)	0.78 (0.64 - 0.96)	1.01 (0.82 - 1.24)	0.68 (0.43 - 1.09)	0.96 (0.79 - 1.15)
Tertile 3	1.05 (0.74 - 1.51)	0.74 (0.61 - 0.90)	0.83 (0.68 - 1.03)	0.79 (0.51 - 1.24)	0.88 (0.72 - 1.07)
**Health status**					
No. of chronic disease(s)^c^					
0	1	1	1	1	1
1-2	1.28 (0.84 - 1.96)	0.97 (0.78 - 1.22)	1.76 (1.41 - 2.21)	1.06 (0.60 - 1.87)	1.01 (0.82 - 1.23)
≥3	2.41 (1.47 - 3.95)	1.44 (1.08 - 1.93)	2.52 (1.89 - 3.37)	1.22 (0.62 - 2.38)	0.95 (0.73 - 1.25)
Self-rated health status					
Good	1	1	1	1	1
Poor	1.63 (1.05 - 2.53)	1.43 (1.15 - 1.79)	2.70 (2.13 - 3.41)	2.51 (1.27 - 4.95)	1.56 (1.27 - 1.92)

^a^Household income was categorized into quartiles based on equalized household income: low (lowest 25%), middle-low (26%−50%), middle-high (51%−75%), and high (highest 25%).

^b^Energy intake tertiles were defined using gender-specific cut-off values: for men, Tertile 1 < 1,573 kcal/day, Tertile 2 1,573–2,120 kcal/day, and Tertile 3 > 2,120 kcal/day; for women, Tertile 1 < 1,182 kcal/day, Tertile 2 1,182–1,615 kcal/day, and Tertile 3 > 1,615 kcal/day.

^c^Chronic diseases include hypertension, stroke, myocardial infarction, angina, osteoarthritis, rheumatoid arthritis, asthma, diabetes mellitus, cancer, and liver disease.

Odds ratios and 95% confidence intervals were calculated by comparing participants with each frailty component to those without the respective component (reference).

Multivariable logistic regression was performed in participants with complete information on covariates (3,643 women).

Estimates for each factor (e.g., age group, education level) were adjusted for all other factors in the table. Analyses were repeated separately for each factor.

Across both men and women, older age was generally associated with higher odds of several frailty components. Overall, higher educational attainment was generally associated with lower odds of key frailty components, particularly weakness and slowness. In men, college or above was associated with lower odds of weakness (OR = 0.65, 95% CI: 0.47–0.90) and slowness (OR = 0.58, 95% CI: 0.42–0.82). In women, high school and college or above were associated with lower odds of slowness (high school: OR = 0.44, 95% CI: 0.32–0.60; college or above: OR = 0.61, 95% CI: 0.39–0.97), and high school was also associated with lower odds of weakness (OR = 0.61, 95% CI: 0.46–0.82). With respect to household income, men in the high-income group had a lower risk of weakness and slowness compared to those in the low-income group. For women, those in the middle-high income group had a lower risk of slowness and low physical activity. Having a larger household size was associated with an increased risk of weight loss in men only (OR = 2.50, 95% CI: 1.02–6.13).

Compared to those who were underweight, both men and women with higher BMI categories showed a tendency toward reduced risk of the weakness component, and in men, higher BMI was also associated with a decreased risk of weight loss. The association between smoking status and frailty components differed by gender. In men, current smoking was associated with an increased risk of low physical activity (OR = 1.50, 95% CI: 1.12–2.00), while in women, current smoking was associated with a higher risk of slowness (OR = 1.98, 95% CI: 1.08–3.62). For alcohol consumption, moderate intake in men was associated with a reduced risk of weakness, slowness, and low physical activity, whereas in women, low alcohol intake was associated with a lower risk of weakness and exhaustion.

Among men, eating breakfast with family was associated with a lower risk of weakness, while eating dinner with family was linked to a reduced risk of weight loss. In women, eating dinner with family was associated with a decreased risk of exhaustion, whereas eating breakfast with family tended to increase the risk of low physical activity. In terms of energy intake, both men and women in the middle intake group (men: 1573–2120, women: 1182–1615 kcal) had a lower risk of weakness compared to those in the low intake group (men: <1573, women: <1182 kcal). Among women, the highest intake group (>1615 kcal) was also associated with lower risk of weakness (OR = 0.74, 95% CI: 0.61–0.90). For men, those in the highest intake group (>2120 kcal) also showed a reduced risk of both weakness and low physical activity. Having three or more chronic diseases was associated with higher risks of weakness, slowness, and low physical activity in men, with the risk for slowness being especially pronounced (OR = 4.32, 95% CI: 2.86–6.55). Among women, the presence of three or more chronic diseases was linked to higher risks of weight loss, weakness, and slowness. Additionally, poor self-rated health was associated with an increased risk of all five frailty components in women.

## Discussion

This study comprehensively examined the associations between frailty and sociodemographic and lifestyle factors by gender among older Korean adults. Increased age, lower educational attainment, lower BMI, a higher number of chronic conditions, and poor self-rated health status were all significantly associated with a greater risk of frailty regardless of gender. Among men, higher household income and greater energy intake, and monthly alcohol consumption were associated with lower odds of frailty, while in women, health-related behaviors such as smoking cessation, more frequent family dinners, and lower breakfast frequency were associated with a reduced risk of frailty.

Various prior studies have consistently reported gender differences in frailty, and the present study corroborates this gender disparity by finding that women have a significantly higher probability of being frail compared to men [26,27]. Similar findings were reported in the Tromsø cohort study in Norway, where the prevalence of frailty was higher among women than men [27]. Additionally, that study demonstrated that mortality rates among frail individuals were higher in men, suggesting that men may experience relatively worse health outcomes once frailty develops. Although the current study did not assess mortality outcomes, it is plausible that analogous mechanisms are at play. These findings underscore the importance of considering sex-specific factors in the prevention and management of frailty, highlighting the need for gender-tailored policies and interventions.

Demographic and socioeconomic factors were also found to be closely related. A common trend was observed in both sexes, whereby advanced age, lower educational attainment, and lower income levels were all associated with an increased risk of frailty. Frailty is a natural phenomenon that occurs with the progression of aging, and accordingly, the proportion of the population in the frail stage was similarly higher with increasing age [26].

A tendency for increased frailty risk was observed among individuals with lower income and educational levels, which may reflect limited awareness and fewer opportunities for prevention and management of frailty [28]. Notably, among men, having at least a high school diploma or higher education was associated with faster walking speed, one of the key components of frailty, suggesting a potential protective effect against frailty. These findings are consistent with previous research, which also reported that both men and women with a university degree exhibited significantly faster walking speeds compared to those without higher education [29]. Collectively, these results indicate that sociodemographic factors, particularly educational attainment, are closely linked to physical function—especially frailty components such as muscle weakness and walking slowness—and may contribute to an increased risk of frailty. This pattern supports the interpretation that frailty reflects the cumulative influence of long-term social and structural disadvantages, rather than being solely an individual-level clinical condition [30,31].

Lifestyle factors were also found to be closely associated with frailty. A common trend was observed in both genders, whereby underweight status, low dietary intake, the presence of three or more chronic diseases, and poor self-rated health were all associated with an increased risk of frailty. In the Mexican elderly population, being underweight was associated with approximately double the risk of frailty compared to normal-weight peers, possibly due to the increased likelihood of muscle mass loss resulting from malnutrition among underweight older adults [32]. Furthermore, men with normal weight, overweight, or obesity had a lower risk of weight loss and muscle strength decline compared to underweight men, and a similar trend was observed in women as well. One study also reported that normal-weight men exhibited faster gait speed than other groups, whereas underweight women demonstrated significantly slower walking speed [29]. These findings suggest that being underweight may act as a key risk factor for frailty via mechanisms such as muscle weakness and reduced gait speed.

The number of chronic diseases was also found to significantly influence the risk of frailty. Individuals with three or more chronic conditions exhibited a significantly higher risk not only of frailty but also of muscle weakness and decreased gait speed in both men and women. A systematic review and meta-analysis targeting community-dwelling older adults similarly identified having three or more chronic diseases as a key risk factor for frailty [26]. Furthermore, Welmer et al. reported that both men and women with two or more chronic conditions had significantly slower gait speeds compared to those with fewer chronic diseases [29]. These findings suggest that multiple chronic diseases may interact with frailty to further increase the risk of adverse health outcomes.

Older adults who evaluated their health status as poor showed a higher risk of frailty and its components in both men and women. In one previous study, participants who evaluated their health status as poor showed a significantly higher risk of weight loss, decreased walking speed, and exhaustion compared to participants who evaluated their health status as good [33]. The results of this study also showed that when the self-assessment of health status was poor, significant odds ratios were observed in all frailty factors in women, and significant results were derived in slowness and low physical activity in men. This confirmed that the risk of frailty and frailty factors increased as the self-assessment of health status was poor. In addition, a previous study reported that women with poorer self-rated health status had higher mortality rates [34]. This suggests that self-assessment of health status is closely related not only to the risk of frailty but also to various health outcomes such as mortality. Therefore, it is necessary to conduct regular self-assessment of health status to effectively reduce the risk of frailty in the elderly. In particular, in the case of women, self-assessment of health status can be used as an important indicator to comprehensively understand women’s health risks, so a policy approach that actively intervenes and prioritizes management seems necessary.

Differences by gender were observed in the association between lifestyle factors and frailty. Among women, smoking and having breakfast and dinner with family were significantly associated with frailty, whereas these associations were not significant in men. Specifically, in this study, smoking was found to increase the risk of frailty in women. In contrast, a previous study reported that smoking had a detrimental effect on frailty primarily in men, with no significant association observed in women [35]. Nevertheless, smoking in women has been reported to negate the survival advantage that women typically have compared to men. Additionally, an increased amount of smoking was associated with higher mortality rates in both men and women, with women exhibiting a greater risk of premature death and stroke [36].

Differences by gender were also observed in the association between eating with family and frailty. While no significant associations were found in men, a contrasting pattern emerged in women. Older women who ate breakfast with their family exhibited a higher risk of frailty, whereas those who ate dinner with their family showed a tendency toward a lower risk. These findings suggest that not only the presence of shared meals but also their timing and social context may differentially influence frailty risk in later life. The increased frailty risk associated with having breakfast with family among women may reflect broader sociocultural and contextual factors, including caregiving burdens, meal preparation stress, or the division of social roles. Previous studies have suggested that shared meals can be embedded within gendered divisions of unpaid work and time pressure, particularly in later life, when caregiving and household responsibilities often remain concentrated among women [37,38]. Time-use research indicates that caregiving and domestic activities frequently peak around mealtimes, especially in the morning, which may coincide with higher levels of role strain and time pressure. Conversely, eating dinner with family may be more likely to occur under relatively lower time constraints and function as opportunities for social interaction and emotional support, potentially contributing to a lower risk of frailty. In previous study conducted in Japan reported that not eating with family was associated with higher frailty risk in both men and women, which contrasts with our findings [39]. Such discrepancies suggest that cultural context may shape how family meals relate to frailty and other health outcomes. Although this study could not directly assess caregiving responsibilities, domestic labor, or perceived burden, these findings highlight the importance of considering the social context and timing of shared meals when interpreting associations between family mealtime practices and frailty. Therefore, further in-depth analyses across diverse countries and cultures is warranted to better understand the complex relationship between family mealtime practices and frailty risk.

In this way, it was confirmed that demographic and lifestyle factors affect frailty and frailty factors. Considering Korea is entering a super-aging society, the need for multifaceted policies to prevent frailty is further emphasized. For example, in the case of socioeconomic factors such as education and income, it is necessary to guarantee lifelong education opportunities even in old age and actively establish support policies for low-income elderly people. Moreover, the consistent vulnerability observed among older adults with lower education and income levels suggests that targeted screening and intervention strategies focusing on these groups may be particularly effective. In addition, since smoking, drinking, and dietary behaviors had different effects on frailty components depending on gender, interventions that account for heterogeneity within older populations—rather than relying on gender alone—may enhance effectiveness.

This study has the following limitations. First, due to the cross-sectional study design, the causal relationship between demographic and lifestyle factors and frailty cannot be clearly established. Because the association between demographic and lifestyle factors and frailty may be bidirectional, future studies are needed to confirm the causal relationship. Second, the use of modified frailty criteria based on proxy measures available in the KNHANES, rather than the original Fried phenotype, may have introduced potential misclassification of frailty status. Third, regarding missing data and selection bias, approximately 32% of eligible participants were excluded because of missing information for at least one frailty component and/or nutrition survey data [22]. This exclusion may have introduced selection bias if missingness was related to frailty status or sociodemographic/lifestyle characteristics; therefore, the observed associations should be interpreted with caution, and future studies using longitudinal data and/or methods that better account for missingness are warranted. Fourth, although we adjusted for major sociodemographic and health-related factors, the possibility of residual confounding from unmeasured variables—such as cognitive function, depression, social support quality, and specific caregiving roles—cannot be ruled out. Fifth, self-reported variables, including dietary intake and perceived stress, are subject to inherent recall bias and measurement error, which may affect the accuracy of the observed associations. Next, separated, divorced, and never married participants were grouped into a single marital status category due to the small number of never married individuals in the sample. While this approach was adopted to ensure statistical stability, these marital status subgroups may differ in their social, economic, and health-related characteristics, and consequently in their frailty risk profiles. As a result, this classification may have limited our ability to fully capture heterogeneity in frailty risk across marital status subgroups. Future studies with larger sample sizes should examine frailty risk using more finely disaggregated marital status categories to better reflect these potential differences. Finally, this study was conducted on Korean elderly people, and there are limitations in generalizing the results to elderly people in other countries with different lifestyles. Nevertheless, this study aimed to clarify the relationship between frailty and lifestyle and sociodemographic factors, and this is significant as basic data for establishing management and prevention strategies for frail elderly people in Korea, which is entering an aging society. Further follow-up studies should be conducted continuously to elucidate the causal relationship between lifestyle and sociodemographic factors and frailty.

## Conclusion

This study comprehensively analyzed sociodemographic and lifestyle factors, including gender differences, influencing frailty and its components among older adults in Korea. Higher educational attainment, adequate body weight, and higher income were positively associated with a reduced risk of frailty in both men and women, whereas the presence of chronic diseases, underweight status, and poor self-rated health increased the risk of frailty components such as slowness and muscle weakness. Additionally, lifestyle factors like smoking and family mealtime patterns showed differential associations with frailty between genders. These findings suggest that educational attainment, as a marker of long-term socioeconomic advantage, may have enduring implications for healthy aging, highlighting the potential benefits of educational and social investments earlier in the life course. At the same time, the strong associations observed between education, income, and frailty in later life indicate the importance of identifying older adults with lower educational and socioeconomic backgrounds as priority groups for prevention and management efforts. Furthermore, the results underscore the need for targeted and effective interventions that address heterogeneity within the older population, including lifestyle modification programs, chronic disease management, caregiving support, and economic assistance, rather than relying on gender alone as a basis for intervention. Accordingly, this study provides valuable practical and policy implications for frailty prevention and health promotion in Korean older adults and serves as a robust reference for the development of customized educational materials targeting diverse subgroups within this population.

## Abbreviations

BMI: body mass index
CIs: confidence intervals
KNHANES: Korean National Health and Nutrition Examination Survey
ORs: odds ratios
SE: standard error.
